# Fabrication and Characterization of Curved Compound Eyes Based on Multifocal Microlenses

**DOI:** 10.3390/mi11090854

**Published:** 2020-09-16

**Authors:** Gaoge Lian, Yongshun Liu, KeKai Tao, Huaming Xing, Ruxia Huang, Mingbo Chi, Wenchao Zhou, Yihui Wu

**Affiliations:** 1 State Key Laboratory of Applied Optics, Changchun Institute of Optics, Fine Mechanics and Physics, (CIOMP), Chinese Academy of Sciences, Changchun 130033, China; lian_gaoge@163.com (G.L.); taokekai18@mails.ucas.ac.cn (K.T.); xhm3653@163.com (H.X.); huangruxia5425@163.com (R.H.); chimb@sklao.ac.cn (M.C.); zhouvc@ciomp.ac.cn (W.Z.); 2 University of Chinese Academy of Sciences, Beijing 100039, China

**Keywords:** compound eyes, microlens array, large field of view, hot embossing, multiple focal lengths

## Abstract

Curved compound eyes have generated great interest owing to the wide field of view but the application of devices is hindered for the lack of proper detectors. One-lens curved compound eyes with multi-focal microlenses provide a solution for wide field imaging integrated in a commercial photo-detector. However, it is still a challenge for manufacturing this kind of compound eye. In this paper, a rapid and accurate method is proposed by a combination of photolithography, hot embossing, soft photolithography, and gas-assisted deformation techniques. Microlens arrays with different focal lengths were firstly obtained on a polymer, and then the planar structure was converted to the curved surface. A total of 581 compound eyes with diameters ranging from 152.8 µm to 240.9 µm were successfully obtained on one curved surface within a few hours, and the field of view of the compound eyes exceeded 108°. To verify the characteristics of the fabricated compound eyes, morphology deviation was measured by a probe profile and a scanning electron microscope. The optical performance and imaging capability were also tested and analyzed. As a result, the ommatidia made up of microlenses showed not only high accuracy in morphology, but also imaging uniformity on a focal plane. This flexible massive fabrication of compound eyes indicates great potential for miniaturized imaging systems.

## 1. Introduction

As an increasingly important optical element, a microlens array (MLA) is characterized by its small size, light weight, and compactability. It provides a universal approach for compact micro-optics systems and is widely used in liquid crystal devices [1,2], laser beam homogenization [3,4], naked-eye 3D displays [5], and artificial compound eyes [6,7,8,9]. In particular, curved artificial compound eyes have generated more attention recently owing to their large field of view and low aberration or distortion. A variety of manufacturing methods have been proposed to fabricate curved artificial compound eyes, including but not limited to laser lithography technology [10], ultra-precision machining [11,12], hydrogel shrinkage [13], two-photon polymerization [14], bottom-up technology [15,16], thermal reflow of two different polymeric materials [17], and membrane deformation of polymers [18] assisted by differential air pressure [19,20,21] or bending ball [22,23,24]. While the fabrication of compound eyes has flourished, the application of components in the system has developed slowly due to a lack of proper detectors. Because the focal plane of arthropod -inspired compound eyes is arranged on a curve instead of a planar substrate, some approaches were used to try to modify the detectors by integrating the detection and microlens arrays [25] or stacking, cutting, and curving the detector planes [26]. Another prevailing strategy was to add an optical relay that steered incident rays and formed images on commercial flat detectors, such as freeform prisms [27,28], sets of lenses [29,30], and optical fibers [19,31]. These approaches successfully associated compound eyes with planar detectors.

However, further extension of the systems was impeded by complicated procedures, alignment accuracy, and tight tolerance. More recently, a one-lens compound eye structure was put forward to replace custom-made detectors and complex systems, by constructing microlenses with different focal lengths at different positions on the curved surface. Thermal reflow [32], molding processes [33,34], and inkjet droplets [35] were used to manufacture MLAs. However, there are concerns about the volume loss during the photoresist melting process, the surface profile quality of the molds [36], and the consistency of microdroplets when printing a dense array one by one. Contactless hot embossing is considered to be a versatile one-step technique to prepare MLAs regardless of the quality of the molds’ inner relief, applied in mass production of microlens arrays [37,38], and the radius of the curvature can also be precisely controlled by process parameters. This method demonstrates the potential of making microlenses with multiple focal lengths.

In this paper, based on previous research, a rapid and accurate method for fabricating multiple focal lengths of compound eyes is proposed by combining photolithography, hot embossing, soft photolithography, and gas-assisted deformation techniques. Microlens arrays with different apertures and the same sag height were prepared by a silicon-based contactless polymer hot embossing method. A cycloolefin copolymer (COC) with transition temperature of 140 °C was used to emboss the microlens structure. Compared with polymethyl methacrylate (PMMA), this material is less likely to produce bubbles in the process of thermal pressure rheology. A polydimethylsiloxane (PDMS) membrane was used as an intermediate mold to convert the plane into a curved surface and a metallic cavity was used to ensure the air pressure difference. After photosensitive resin casting and ultraviolet (UV) curing, resinous compound eyes with multifocal microlenses in a circular arrangement on one curved surface were obtained. The geometrical morphology and optical properties were characterized and an imaging contrast experiment was carried out with a plane detector.

## 2. Design and Fabrication

**Figure 1:**
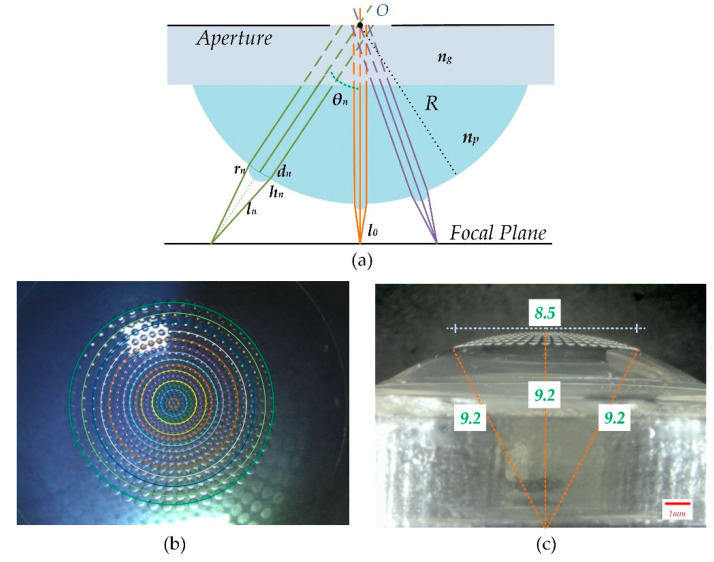
(**a**) Design of microlenses with multiple focal lengths on a curved surface; (**b**) image of curved compound eyes captured by optical stereoscopic microscope; (**c**) lateral view of curved compound eyes captured by digital microscope with large depth of field.

As shown in Figure 1a, multifocal compound eyes located on a planoconvex substrate were designed. O is the optical center of the compound eyes, and the optical ray of each cycle of the MLA passes through the point of *O* due to the aperture stopping. The microlens on the surface apex as the center is denoted as the 0th cycle, and the MLAs extended outward with the circumference are named the 1st, 2nd, *n*th cycles in turn. The microlenses in each annulus cycle possess the same parameters. The distance of the nth microlens along the optical axis between the image plane is $l_{n}$. In the arrangement of gradually increased cycles from 0th to 16th, the focal length of every microlens array must be resized following $l_{n}$, so that the images formed by different arrays can fall on the same planar detector. We set the vertical distance between the 0th cycle microlens and the image sensor plane as $l_{0}$, which is equal to the focal length of the 0th microlens. In particular, the refractive index of substrate holding up the compound eyes is recorded as $n_{g}$, but is not limited to glass. In this simulation model, the small eye is treated as a thin lens, and according to the thin lens equation and geometrical relationships, three equations can be deduced:

(1) $$l_{n}=f_{n}=\frac{r}{1-n_{p}}$$

(2) $$r_{n}=\left(1-n_{p}\right)\left[\frac{R+h_{0}+l_{0}}{\cos\theta_{n}}-\left(R+h_{0}\right)\right]$$

(3) $$d_{n}=\sqrt{r_{n}{}^{2}-{\left(r_{n}-h_{n}\right)}^{2}}$$

where $d_{n}$ is the diameter of the *n*th cycle microlens, $h_{n}$ is the altitude of the microlens in the *n*th cycle, and $n_{p},\;R$ represent the refractive index and curvature radius of the polymeric compound eyes. The angle between the optical axis of the *n*th microlens and the primary optical axis is written as $\theta_{n}$. The index of the substrate holding up the compound eyes is recorded as $n_{g}$, which is close to the compound eyes to avoid the refraction between them. As shown in Figure 1c, the compound eye and its substrate form a hemispherical shape, the height and radius of the plano-convex substrate are 9.2 mm. All of these ensure that the incident light through the aperture incident propagate along the optical axis of the microlenses on the curved surface. 

**Figure 2:**
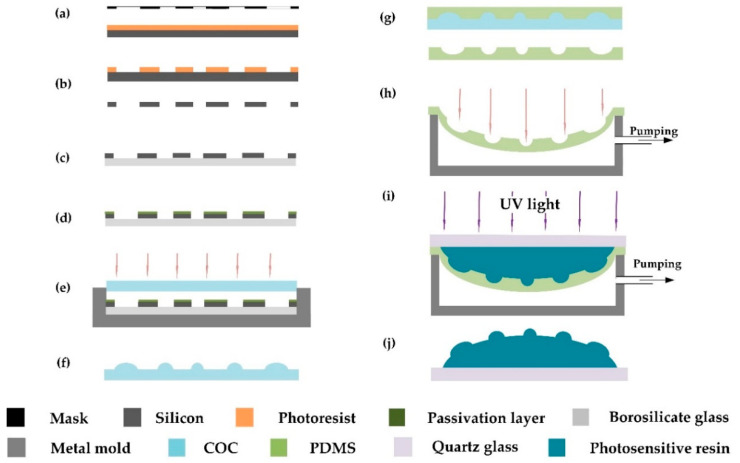
Schematic of manufacturing curved compound eyes: (**a**) AZ5214 photoresist spin-coated on silicon substrate and exposed; (**b**) developed and etched of silicon wafers; (**c**) patterned silicon substrate bonded to borosilicate glass; (**d**) passivation layer grown on the surface; (**e**) polymer heated and pressed; (**f**) polymer peeled off to get microlens array (MLA) pattern; (**g**) structure replicated on polymer with polydimethylsiloxane (PDMS); (**h**) PDMS membrane deformed and packaged in a sealed cavity under pressure difference; (**i**) photosensitive adhesive poured and cured; (**j**) curved compound eyes peeled off.

Figure 2 illustrates the fabrication of curved compound eyes. Specifically, the standard photolithography process was adopted to transfer the mask patterns onto the silicon wafer, and a double-sided ultraviolet (UV) mask aligner (Karl Suss, MA6/BA6, Munich, Germany) was used for soft contact exposure. Then, an inductively coupled plasma etching machine (Alcatel, A601E, Annecy, France) was used to etch the silicon wafer at a speed of 8 μm/min for 6 min. The Si mold with arrays of micropores with different diameters was obtained by removing the residual photoresist. Then a fluorine-based passivation layer was grown on the surface of the mold to facilitate subsequent demolding. A polymer of COC with a thickness of 500 μm was pressed under pressure of 200 mbar at 145 °C for 6 min by a Suss bonding system (Karl Suss, SB6E). After purging with nitrogen gas for 10 min and cooling to 80 °C, the polymeric MLAs with different focal lengths were demolded.

**Figure 3:**
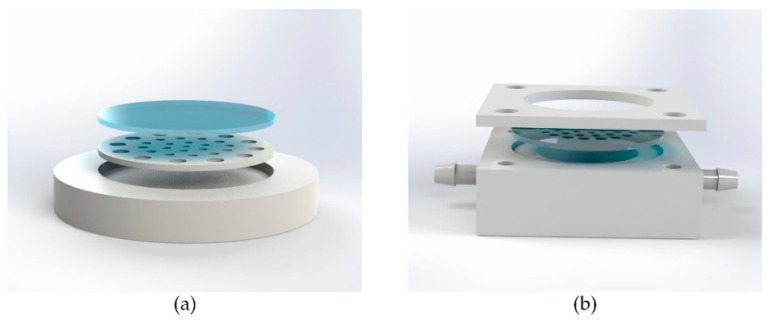
Three-dimensional diagram of the home-built metal mold structure: (**a**) grooved tray for relieving excess pressure in the hot embossing process; (**b**) sealed cavity for package of the PDMS intermediate die with MLA structure.

**Figure 4:**
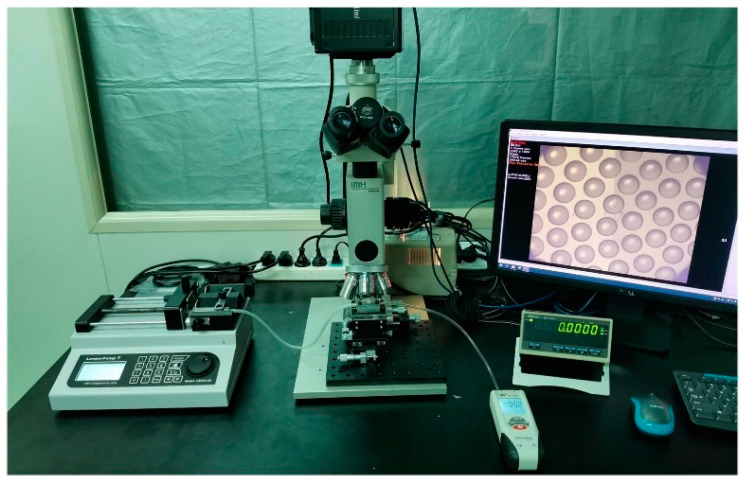
Experimental setup used to measure altitudes of MLAs with different diameters and sag height of PMDS deformation under different pressure in real time.

Soft lithography and gas-assisted deformation were applied in the transformation of planar MLA structure from plane to surface. PDMS (Dow Corning, Sylgard 184, Midland, MI, USA) was used as the elastomeric die in this process. The curing agent and PDMS prepolymer were mixed at a weight ratio of 1:10 and degassed in a vacuum oven, then the mixture was poured onto the polymeric MLAs and cured at 80 °C for 2 h. Then the PDMS membrane was peeled off from the polymeric MLA structure and put into a sealed cavity. As shown in Figure 3b, the two holes on both sides of the cavity were used to control the pressure difference by a digital syringe pump (RISTRON RSP04-C). The value of pressure difference was detected and displayed in real time by a digital manometer (Hti HT1891) (Figure 4). A photosensitive resin (Norland Products, NOA63, Cranbury, NJ, USA) was then cast onto the deformed membrane and cured under an ultraviolet (UV) light with a wavelength of 365 nm at an irradiance intensity of 75 wm/cm^2^ for 90 s. Finally, the curved compound eye with multifocal microlenses was obtained successfully within 4 h. Replicas of the compound eye can be manufactured in 2 min.

## 3. Result and Discussion

In the process of hot embossing, some measures were taken to ensure repeatability of the Si mold and the uniformity of the MLAs. The silicon wafer was completely bonded to 1 mm thick borosilicate glass to increase the hardness of the mold. In addition, we reserved sufficient time before hot embossing at 130 °C for 10 min for heat conduction and stress relief because of the difference of heat conduction in the embossing chamber and the non-uniform distribution of internal stress. Moreover, in order to avoid redundancy and uneven distribution of pressure, we placed the silicon mold and COC into a grooved tray (Figure 3a), and the depth of the groove was slightly lower than the total thickness of the stack.

**Figure 5:**
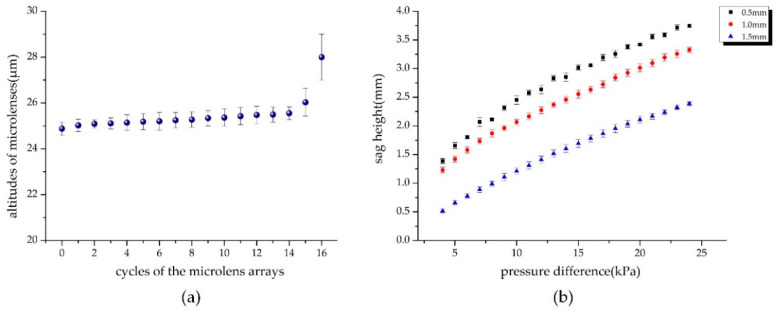
Result of morphology measurement experiment: (**a**) altitudes of pressed polymeric MLAs with different diameters from 0th to 16th cycles; (**b**) deformation of PDMS membranes with different thickness under pressure from 4–24 Kpa.

The altitudes of plane microlenses and the sag height of the curved surface were measured by a non-contact depth measurement microscope unit (Union Optical IMH). To achieve a more reliable result, we took the average of many measurements and plotted the results with error bars (Figure 5).

As shown in Figure 5a, there was good uniformity in the altitudes of the microlenses from the 0th to 14th cycles, but the deviation started to increase from the 15th, which was affected by the filling efficiency. Since the fill factor of the micropores decreased sharply from the 15th cycle, the polymer was more sufficiently filled, so the altitudes of the microlens arrays in the 15th and 16th cycles were larger than the inner ones. Despite the 16th cycle, the altitudes of the polymer MLAs did not change much with the increasing diameters from the 0th to 15th cycles, and the deviation was less than 6%. Figure 5b illustrates that under the same pressure, the smaller the membrane thickness, the greater the sag depression, that is, the more sensitive the response to pressure changes. Our previous studies showed that the elastic deformation of PDMS membrane is linear to the pressure difference in a certain range [39], and Figure 5b shows that there was a wider range of linear deformation and relative morphological stability when the thickness was 1.0 mm. We finally set the membrane thickness as 1.0 mm. The diameters of MLAs in different cycles based on the polymer and PDMS were also observed by an ultra-depth-of-field microscope (KEYENCE, VHX-1000, Osaka, Japan), and the maximum standard deviation was from the MLAs with PDMS. The diameter reduction was about 3% compared to the ideal results, mainly due to shrinkage after PDMS curing and demolding. The results showed relatively high uniformity and accuracy of geometry in the process of deformation.

**Figure 6:**
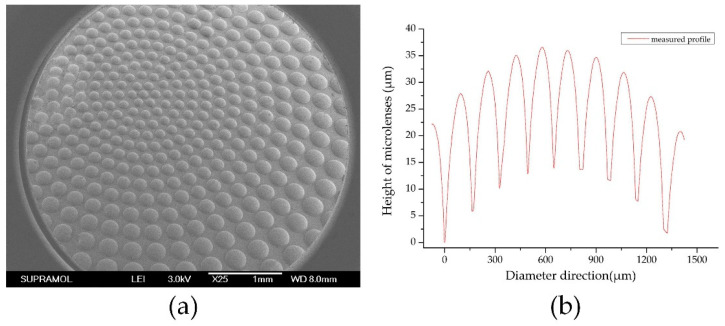
Three-dimensional (3D) profile of the compound eye: (**a**) SEMimage of the multifocal compound eye at side view; (**b**) the measured profile of microlenses in center of the multifocal compound eye.

In particularly, it must be mentioned that the interferometric technique is the most popular and accurate method to assess the presence and the amount of aberrations in an optical system, and the interference microscopy testing allow to perform the full characterization of each kind of microlenses as an accurate procedure [40,41]. Herein, because of the range limitation of our measuring tools, a probe profiler (Veeco, Dektak150, New York, NY, USA) and a scanning electron microscope (SEM; JEOL, JSM-6700F, Tokyo, Japan) were applied to characterize the 3D surface profile of the planoconvex compound eye and microlenses. As shown in Figure 6, the diameter and height of the microlenses on the center areas are approximately 150 μm and 25 μm.

**Figure 7:**
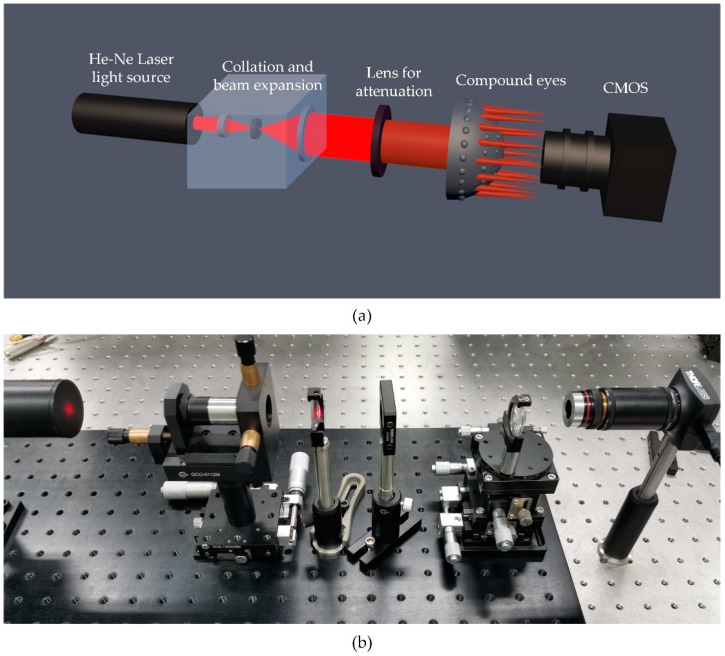
Experimental installation for optical performance: (**a**) schematic diagram of the setup; (**b**) actual platform comprised of He-Ne laser source, microscopic objective lens, pin hole, convex lens, MLAs, optical density filters, microscopic objective lens, and photodetector.

As shown in Figure 7, the collimating and parallel beam transmitted through the MLAs and formed spots on the detector. The spots captured by the detector were output into a displayer, then we adjusted the distance between the MLAs and the detector to form sharp and bright spots, which were considered as the focal spots of these cycles, and noted the position of the MLAs on a two-axis manual stage. MLAs were then moved until the apex surface of the polymer microlens could be seen clearly on the screen, and we recorded the scale of this position. We regarded a single microlens as a thin lens, so its focal length was the distance from the surface to the focal spot, which equaled the difference between the two position coordinates. Deviation of approximately 5% was measured in the testing experiments, mainly due to variations of PMDS membrane in the conversion from plane to surface.

**Figure 8:**
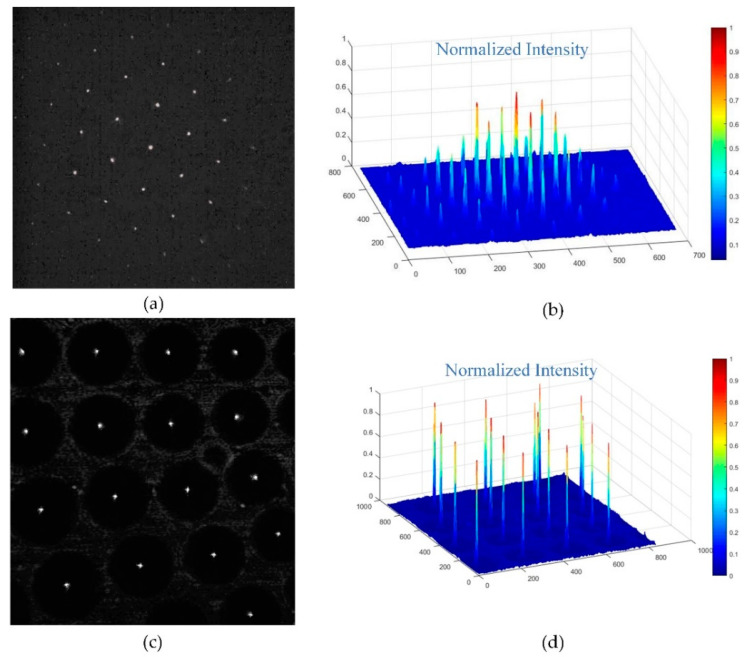
Focal spots of compound eyes with single and multiple focal lengths: (**a**) focal spots of single focal length microlenses on dome area; (**b**) normalized intensity distribution of single focal length microlenses; (**c**) focal spots of multiple focal length microlenses on the middle area; (**d**) normalized intensity distribution of multiple focal length microlenses.

**Figure 9:**
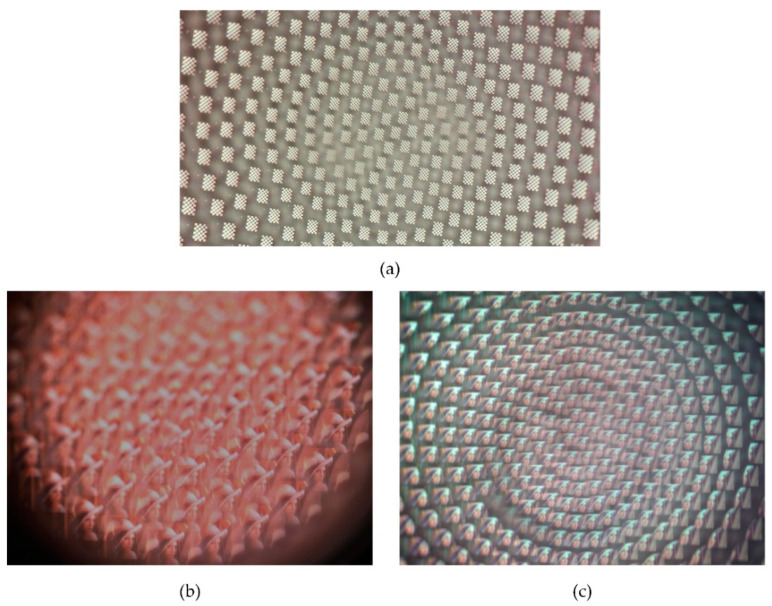
Images captured by the compound eyes: (**a**) calibration plate captured by the multi-focal compound eyes; (**b**) image of Lenna in contrast to the single focal compound eyes; (**c**) image of Lenna captured by the multifocal compound eyes.

Additionally, we took the focal spot images of the compound eyes contrasting single with multiple focal lengths and analyzed the intensity of energy distribution of the spots to verify the uniformity of the microlenses. As shown in Figure 8, the focal spot intensity of single focal length microlenses had good uniformity in cycles alone, but the intensity of multiple focal length microlenses in different cycles was relative homogeneous even in the margin region. The imaging capability of the curved compound eye was also investigated with a commercially available complementary metal-oxide semiconductor (CMOS; Aptina, MT9J003, Boise, ID, USA). A calibration graph with a 6 × 8 black-and-white chessboard and a picture of Lenna were displayed on a screen and then captured by the setup. As shown in Figure 9a, the black and white vertical lines of the chessboard were all orthogonal, indicating that there was less distortion in the image formed by the curved compound eye. Comparing the image of Lenna captured by the single focal compound eye with a focal length of 1.2 mm (Figure 9c), nearly every ommatidium of the compound eye with multifocal MLAs formed clearly and sharply on the CMOS (Figure 9c), proving that all focal spots of the multifocal compound eye were adjusted to one detector plane. These results confirmed the agreement of the experimental samples with the designed ones and the excellent optical performance of the artificial curved compound eye in reducing distortion and defocusing.

**Figure 10:**
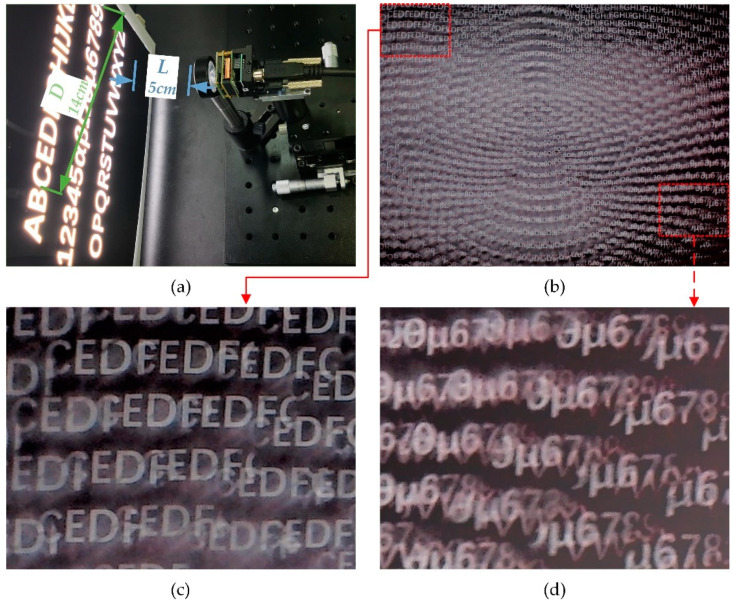
Experiment of field of view (FOV) testing: (**a**) experimental setup of the testing system; (**b**) numbers and letters captured by multifocal compound eyes; (**c**) close look at the upper-left portion of the image; (**d**) close look at the lower-right portion of the image.

The field of view (FOV) testing experiment of the compound eye is shown in Figure 10a. The FOV measurement setup consisted of an optical imaging system, including the compound eye, CMOS, optical displacement stage, and a displayer with a white light-emitting diode (LED) light source as a background and a film with continuous alphabets and numbers. The FOV of the compound eye can be calculated with the following formula:

(4) $$FOV=2\arctan\frac{D}{2L}$$

where *L* is the distance between the compound eye and the screen and *D* is the length of longest diagonal between the two characters captured by the CMOS, which were labeled in blue and green. According to the experimental results exhibited in Figure 10a, the FOV of the curved compound eye can reach approximately 109°. Because the sensor size of this CMOS was 1/2.3 inch (4:3) and the diagonal of the sensor was 7.9 mm, which was smaller than the effective area of our artificial compound eye shown in Figure 1c and Figure 6a, some information transferred from the outermost microlenses could not be received by the CMOS. While there was a reduction of sensor size, the actual FOV of the compound eye was larger than the result observed by the imaging system.

## 4. Conclusions

In summary, we successfully manufactured curved microlens arrays with multiple focal lengths by means of hot embossing, soft lithography, and gas-assisted deformation. A total of 581 microlenses were fabricated on a curved surface within four hours, representing great geometric uniformity and optical performance adapted to the imaging system. Distortion caused by defocusing on the edge was solved well through the multifocal design. The focal lengths ranged from 0.23 mm to 2.09 mm and the field of view of the system achieved 109°. Since the images formed by some marginal areas of the microlens exceeded the pixel area of the detector, by using a CMOS with a larger sensor size and increasing the scale of the microlens arrays, the FOV of the compound eyes could theoretically reach close to 180°. Moreover, accurate aberrations of the single optical elements will be evaluated by interferometric technique to certify the aberrations by the fabrication process in our following study. The one-lens compound eye with massive multifocal microlenses shows great potential to be integrated into micro-opto-electro-mechanical systems, including but not limited to those in motion and medicine imaging, security, and military monitoring.
